# Intuition or Deliberation? The Effects of Decision-Making Modes on Adolescents’ Honest Behaviors: The Moderating Roles of Honesty Tendencies and Victim Situations

**DOI:** 10.3390/bs15111535

**Published:** 2025-11-11

**Authors:** Haowen Yin, Honglai Zhang

**Affiliations:** 1 Kangda College, Nanjing Medical University, Lianyungang 222000, China; yinhaowen@njmu.edu.cn; 2 Faculty of Psychology, Tianjin Normal University, Tianjin 300380, China

**Keywords:** honest behavior, decision-making mode, victim situation, honesty tendency

## Abstract

An ongoing controversy exists regarding whether honest behaviors are driven by intuition or deliberation. To reconcile opposing research viewpoints, this study, grounded in the social heuristic hypothesis, focuses on two key factors that influence honest behaviors: decision-making situations and personal traits. It explores the effects of intuitive and deliberate decision-making modes on adolescents’ honest behaviors and the moderating effect of honesty tendencies and victim situations. A mixed three-factor experimental design was employed, using a “spot-the-difference” task to assess adolescents’ honest behaviors. The results revealed that, in victimless situations, promoting intuitive and deliberate decision-making was more conducive to the honest behaviors of adolescents with high- and low-honesty tendencies, respectively, while in victim situations the effect of decision-making modes on honest behaviors tended to be consistent between individuals with high and low honesty tendencies. Adolescents with high and low honesty tendencies demonstrated more honest behaviors in the intuitive decision-making mode. These findings indicate that the effect of decision-making modes on honest behaviors is a dynamic process of individual–situation co-shaping, emphasizing the significant situational heterogeneity of—and providing a new perspective to improve—adolescents’ honest behaviors.

## 1. Introduction

Honesty, as a universal moral principle in human society, occupies a unique position in the cultivation of adolescents’ core values (Timpau, 2015). However, adolescents frequently exhibit dishonest behaviors (Gordon-Hecker et al., 2024), including cheating on examinations, plagiarizing assignments, concealing facts, and fabricating false information (Malti et al., 2021). These behaviors not only damage their moral reputation but also erode the cornerstones of social fairness and justice. Studies have found that, on average, individuals lie one to two times every day (Hart et al., 2019; Markowitz, 2021). However, adolescents exhibit a significantly higher incidence of dishonest behaviors than do children and adults (Guo et al., 2023). Notably, with increasing age, adolescents’ honest behaviors reveal a fluctuating trend of rising and then falling. The level of honesty in middle and late adolescence (approximately 15–19 years old) is relatively low (Kang & Fu, 2021; F. F. Zhang et al., 2022). Moral cognition and behavioral patterns from this period profoundly affect morality in adulthood (Malti et al., 2021). Hence, it is important to explore the intrinsic cognitive mechanisms of honest behaviors in adolescents to develop targeted intervention strategies and contain dishonest behaviors effectively.

Adolescents’ honesty is affected by their internal decision-making mode. The dual-process theory of decision-making posits that individual behavioral decision-making involves two different modes: intuitive decision-making—which is relatively fast, with less demand on cognitive resources—and deliberate decision-making—which is relatively slow, with greater demand on cognitive resources (J. S. B. T. Evans & Stanovich, 2013). Researchers have manipulated decision-making modes by limiting reaction times or cognitive resources (Bago & De Neys, 2017; Cruyssen et al., 2020; Reis et al., 2023; X. M. Wang et al., 2022). The results consistently reveal that under time pressure, high cognitive load, and high ego depletion, individuals are more likely to activate their intuitive decision-making mode, with their behavioral decisions being influenced by intuition. Contrarily, conditions of time delay, low cognitive load, and low ego depletion are more likely to activate individuals’ deliberate decision-making mode, with their behavioral decisions being influenced by deliberate rational analysis (A. M. Evans et al., 2015; Rand, 2016; Xiong et al., 2021).

Grounded in dual-processing theory, researchers have proposed two competing honesty hypotheses: the “Grace” hypothesis and the “Will” hypothesis (Greene & Paxton, 2009; Li et al., 2022). The former contends that honest behaviors result from an individual’s intuitive automatism and that the intuitive decision-making mode is more conducive to honest behaviors (Greene & Paxton, 2009; Haidt, 2001). The latter contends that honest behaviors result from an individual’s conscious cognitive control and that the deliberate decision-making mode is more conducive to honest behaviors (Greene & Paxton, 2009; Shalvi et al., 2012). Both hypotheses have been substantiated with empirical evidence. Studies supporting the “Grace” hypothesis have revealed that high time pressure promotes honesty (Capraro, 2017; Capraro et al., 2019). Compared with lying, honest responses are faster and take less time to produce (Foerster et al., 2019; Suchotzki et al., 2017; X. M. Wang et al., 2022). Similarly, when individuals are in a state of high ego depletion or cognitive load (weakened rational control ability), their behaviors tend to be more honest under intuition (Bereby-Meyer et al., 2020; Reis et al., 2023). However, studies supporting the “Will” hypothesis present contradictory evidence. Under high time pressure, individuals are more likely to engage in dishonest behaviors (Shalvi et al., 2012; Köbis et al., 2019; Hausladen & Nikolaychuk, 2024). In the intuitive decision-making mode induced by high depletion or cognitive load, individuals exhibit more dishonest behaviors (Fan et al., 2022; Y. Wang et al., 2024). Notably, despite the seemingly contradictory conclusions, their manipulation paradigms are similar because previous studies have ignored the role of key moderating factors (such as situational variables and individual differences). To integrate existing contradictions, researchers proposed the social heuristic hypothesis (SHH).

The SHH emphasizes that past experiences shape individuals’ intuitive responses (Rand et al., 2016). It proposes that some successful and favorable behavioral strategies—such as honesty, helpfulness, and cooperation—become internalized as intuitive responses. Consequently, individuals automatically apply them when making social decisions in different social situations (Rand et al., 2016; Rand & Epstein, 2014). However, the behavioral strategy that becomes a favorable intuitive response for an individual depends on a combination of individual traits and context. Honesty is not the default intuitive response for all individuals; some internalize deception instead. Although this hypothesis provides a new explanation of previous research controversies, the evidence is relatively scarce. Few studies have validated whether the SHH can explain the inconsistency of honest behavioral outcomes in terms of a single dimension of situational or individual factors (Köbis et al., 2019; Pitesa et al., 2013; Speer et al., 2020). Research attention on the effects of the interaction between individual and situational factors on decision-making modes and honest behaviors is lacking. Honest behaviors result from a combination of decision-making situations and the decision-maker’s characteristics. They are largely contingent on the decision-maker’s own honesty tendency, which may therefore moderate the relationship between decision-making modes and honest behaviors. Meanwhile, honesty or deception often occurs in interpersonal interactions. One’s lies affect not only their own earnings but also those of others. Thus, victim situational factors play a crucial role in decision-making patterns and honest behaviors.

First, honest behavior is largely influenced by the individual characteristics of the decision-maker. The HEXACO (Honesty–humility, Emotionality, Extraversion, Agreeableness, Conscientiousness, and Openness to experience) personality structure model’s honesty–humility dimension explains individual differences in dishonest behaviors (Klein et al., 2020). Studies have concluded that individuals with high honesty–humility pay more attention to the interests of others, pursue fairness and justice, and have higher moral standards (Ashton et al., 2014). In contrast, those with low honesty–humility are greedy, cunning, and pay more attention to personal interests (Zettler et al., 2020). Studies have found that honesty–humility significantly and positively predicts moral behavior in both adults and adolescents (Hilbig & Zettler, 2015; Klein et al., 2020). From the perspective of the SHH, individuals with high honesty–humility can be inferred to automatically adopt honesty as their default response (Hilbig, 2022). Consequently, their intuitive decision-making leads to more honest behaviors in daily social interactions. Conversely, individuals with low honesty–humility prioritize self-interest and adopt lying for greater gain as their default response (Heck et al., 2018). Thus, deliberate decision-making paradoxically yields more honest behaviors in this group. Therefore, the influence of decision-making modes on honest behaviors differs between individuals with high and low honesty tendencies. Notably, most previous studies on the classification of individuals with high and low honesty tendencies were screened using the HEXACO’s honesty–humility scale (Guo et al., 2023; Hilbig, 2022; Klein et al., 2020). However, because of the high social desirability of honest behaviors, responses to the scale may exaggerate the true level of individuals’ honesty. Other researchers have categorized participants into high or low honesty groups according to their frequency of deception in experimental tasks (Speer et al., 2020, 2021). However, this measured only short-term, state-based differences in honesty. Therefore, more rigorous subject-screening methods are required to ensure the validity of the results of future studies.

Simultaneously, situational factors also affect honest behaviors. Studies have revealed that the presence or absence of victims (victim situation) in the consequences of dishonesty moderates the relationship between decision-making modes and honest behaviors (Köbis et al., 2019; Pitesa et al., 2013). Pitesa et al. (2013) found that the intuitive decision-making mode promotes more honest behaviors when deception causes harm to others but promotes more dishonest behaviors when deception involves only self-interest. However, this study only considered the role of victim situational factors, overlooking the effect of individual default honesty tendencies. In a meta-analysis of behavioral studies on lying, Köbis et al. (2019) found that promoting intuition leads to more lying when dishonesty harms abstract others and that the intuitive decision-making mode promotes more honest behaviors when dishonest behaviors harm concrete others. However, the relatively unbalanced sample distribution of studies using concrete and abstract victims in this meta-analysis may have biased the results. Therefore, how honesty tendency and victim situations interact to influence the relationship between decision-making modes and honest behaviors remains unclear. Clarifying this interactive mechanism will provide an important basis for understanding the internal cognitive processes underlying honest behaviors.

To sum up, this study aims to examine mid–late adolescent groups from the perspective of individual–situation interaction, focusing on the effect of decision-making modes on the honest behaviors of adolescents with different honesty tendencies in victimless situations (involving only self-interest) and victim situations. Specifically, to guarantee higher experimental ecological validity, Pre-Experiments 1 and 2 are conducted on the optimal duration of picture presentation and the limited duration of time pressure in the experimental paradigm (spot-the-difference task), measuring participants’ honest behaviors. The formal experiment adopts a mixed three-factor experimental design and screens participants with high and low honesty tendencies based on their individual personality traits and short-term behavioral decision-making. Intuitive and deliberate decision-making modes are induced by time constraint and cognitive load, and different self–other benefit methods are used to set up victim and victimless situations. The deception rate of the participants in the spot-the-difference task is used as an indicator of honest behaviors.

Based on previous findings, the hypotheses of this study are as follows: First, in victimless situations, the effect of decision-making modes on honest behaviors differs between adolescents with high and low honesty tendencies. Those with low honesty tendency engage in more honest behaviors by promoting deliberate rather than intuitive decision-making, whereas those with high honesty tendency engage in more honest behaviors by promoting intuitive decision-making (H1). Second, in victim situations, the effect of decision-making modes on honest behaviors tends to be consistent between adolescents with high and low honesty tendencies, potentially evoking more honest behaviors under the intuitive decision-making mode (H2).

## 2. Pre-Experiments

### 2.1. Pre-Experiment 1: Determination of the Presentation Time of the Images in the Spot-the-Difference Task

This study used the “spot-the-difference” task developed by Speer et al. (2020) to measure participants’ (dis)honest behaviors. This task represents a relatively new research paradigm in honesty research. Its highly covert design allows participants to autonomously choose honesty or deception on each trial. Consequently, it effectively examines differences between and within individuals concerning honest behaviors. Because the participants of Speer’s study were foreign adults (aged 18–35 years), and previous studies have revealed differences in response speed and reaction time between adults and adolescents in visual search tasks (Stavropoulou & Stavropoulos, 2020), to ensure that Chinese adolescent participants found differences between images in a reasonable amount of time and to reduce operational errors (false positives), this study conducted two pre-experiments that established the optimal duration of image presentation and the basic duration of time pressure in this paradigm.

#### 2.1.1. Experimental Purpose

To investigate the time required for Chinese adolescents to find differences between images in the “spot-the-difference” task.

#### 2.1.2. Participants

Forty adolescents (19 boys and 21 girls) aged 15–19 years (*M* ± *SD* = 16.73 ± 1.01 years) were randomly selected from a middle school in Lianyungang City, Jiangsu Province. Before the experiment, their parents or legal guardians were informed of the purpose, process, and time of the experiment. After obtaining the parents’ or legal guardians’ consent, participants were asked to sign an informed consent form. All participants volunteered to participate in the experiments. They had normal or corrected visual acuity, self-reported no other mental disorders, and were right-handed. After completion of the experiment, they were paid accordingly. All experiments were approved by the Ethics Review Committee of Tianjin Normal University.

#### 2.1.3. Materials and Procedure

**Figure 1:**
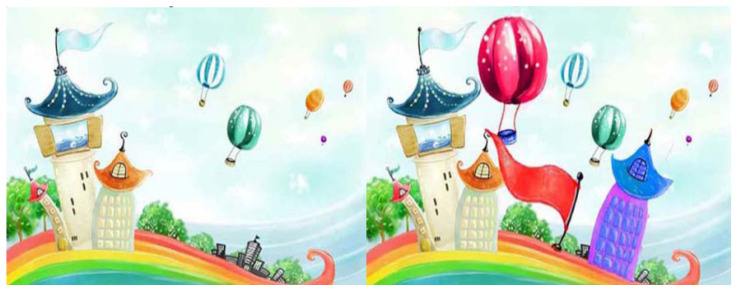
Example of the “spot-the-difference” task. Note: the image is from “To cheat or not to cheat: Cognitive control processes override our moral default (Version 1),” by (Speer et al., 2020), Erasmus University Rotterdam (EUR). CC BY 4.0.

The image library created by Speer et al. (2020) was used for this study. Cartoon images of landscapes were selected to make them engaging and challenging for the participants. Landscapes were chosen because they typically meet the necessary criteria of including several different objects. The stimuli comprised a pair of identical images and a specific number (1–3) of different images created using Photoshop. The differences comprised adding or removing objects from landscape images or changing their colors. In this experiment, 172 image pairs were selected as experimental materials, of which 6 pairs were used as practice trials and 166 pairs were used as formal trials. Each image pair had three differences between them (Figure 1). The experimental program was written using E-Prime 3.0.

When they arrived at the laboratory, participants were informed that the purpose of the experiment was to evaluate the difficulty of the images in the “spot-the-difference” task. The experimental task was to find the three differences in each image pair. The procedure was as follows: 500 ms fixation points appeared first, following which an image pair was presented side-by-side on the screen. The participants had to click on the right picture quickly and accurately within 15,000 ms to find the three differences. If they failed to do so, the screen displayed the next image pair. The experiment recorded the reaction time for each click, which gauged whether the participants could find all the differences in the image pairs and how long it took.

#### 2.1.4. Results and Discussion

Based on the choices of 40 participants, 16 image pairs that took too long (more than 15,000 ms) or were too easy to find (less than 2000 ms) were deleted, resulting in 144 pairs of images. With regard to the remaining pairs, 95% of the participants found all three differences in less than 4000 ms (*M* ± *SD* = 3286 ± 683 ms). This high success rate indicates that the ambiguity of the differences in images was low, thus reducing the probability of false positives. The results also suggest that the difficulty levels between the images were similar and that this task was applicable to Chinese adolescents.

In Pre-Experiment 1, the participants were required to click on the specific locations of the three differences. However, in the formal experiment, the participants only needed to perform visual scanning. Therefore, combined with the results of Pre-Experiment 1, the final setting of the image presentation time was 4000 ms, which was sufficient for more than 95% of the participants to find the three differences.

#### 2.1.5. Conclusions

For Chinese adolescents, the image presentation time in this study was set to 4000 ms in the “spot-the-difference” task.

### 2.2. Pre-Experiment 2: Determination of the Duration of Time Pressure in the Spot-the-Difference Task

#### 2.2.1. Experimental Purpose

To determine the basic duration of time pressure for adolescents in the “spot-the-difference” task.

#### 2.2.2. Participants

Forty-five adolescents (22 boys and 23 girls) aged 15–19 years (*M* ± *SD* = 17.27 ± 1.23 years) were randomly selected from a middle school in Lianyungang City, Jiangsu Province. The recruitment procedure, inclusion criteria, and consent and ethical approval processes were the same as in Pre-Experiment 1.

#### 2.2.3. Materials and Procedure

When they arrived at the laboratory, participants were informed that the objective of the experiment was to examine individual visual search characteristics. The task was to identify the three differences between each image pair. They only needed to spot differences, without having to indicate their specific locations. The procedure was as follows: a fixation point appeared for 500 ms, then an image pair was displayed on the screen. The participants were asked to perform a visual search within 4000 ms. The screen then displayed the question, “Did you find three differences? If yes, press the ‘F’ key; if no, press the ‘J’ key.” The computer recorded participants’ keypress responses without time constraints. Five practice trials and 144 formal experiments were conducted in total. The experimental program was written using E-Prime 3.0.

#### 2.2.4. Results and Discussion

The average response time of the participants under no time constraint was 2461 ms (*SD* = 338 ms). Prior research established 50% of the average decision time without time constraints as a criterion for setting time pressure (Weenig & Maarleveld, 2002; X. M. Wang et al., 2022). Based on this criterion, the time pressure to make a decision as quickly as possible in this study was set to within 1200 ms. The time delay was set to at least 3000 ms to ensure participants’ careful consideration and allow for sufficient time to make deliberate decisions (Yang & Chen, 2020).

Pre-Experiment 2 mainly determined the basic duration of time pressure for adolescents in the “spot-the-difference” task. Dual-processing theory assumes that intuitive decision-making processes are relatively faster than deliberate decision-making processes (J. S. B. T. Evans & Stanovich, 2013). However, it provides no absolute criterion to define an intuitive response, such as faster than X seconds (Bago & De Neys, 2017). Decision times (2000 ms–10,000 ms) have often been directly limited in previous studies (Capraro, 2017; Mischkowski et al., 2018). However, differences in experimental tasks and materials may result in different decision times among participants. Previous research has revealed that limiting the response time window can make participants rely on intuition to make decisions because time pressure diminishes their reflective ability (Capraro et al., 2019; Cruyssen et al., 2020). Conversely, when participants were asked to contemplate before making a decision, they were more influenced by deliberate analysis and relied on rationality (A. M. Evans et al., 2015; Rand, 2016). Therefore, participants can effectively activate the intuitive decision-making mode when they feel time pressure and urgency in the behavioral decision-making process.

In summary, this study used 50% of the average decision time without time constraints as the standard for time pressure, ensuring the effectiveness of the manipulation of decision-making modes during the experiment (Weenig & Maarleveld, 2002; X. M. Wang et al., 2022). Thus, we hypothesized that participants’ choices were driven more by the intuitive decision-making mode in the time-pressure condition and more by the deliberate decision-making mode in the time-delay condition.

#### 2.2.5. Conclusions

In the “spot-the-difference” task, time-pressure decision-making required participants to make behavioral responses within 1200 ms; time-delay decision-making required participants to think for at least 3000 ms before making a choice.

## 3. Formal Experiment

### 3.1. Experimental Purpose and Assumptions

The formal experiment aimed to investigate the influence of decision-making modes on the honest behaviors of adolescents with high and low honesty tendencies in situations with and without victims.

The following assumptions were made:

(1) In victimless situations, adolescents with low honesty tendency exhibited significantly less honest behaviors in the intuitive than in the deliberate decision-making mode, whereas adolescents with high honesty tendency exhibited significantly more honest behaviors in the intuitive than in the deliberate decision-making mode.
(2) In victim situations, adolescents with high and low honesty tendencies both exhibited more honest behaviors in the intuitive than in the deliberate decision-making mode.

### 3.2. Methods

#### 3.2.1. Participants

The experiment employed G*Power 3.1 (Faul et al., 2007) for sample size estimation. Based on a three-factor mixed experimental design, the total sample size for the 80% level of statistical power at the significance level of α = 0.01 with a medium effect (*f* = 0.25) was 34, with 17 participants in each group. To ensure strict control of the variables and experimental manipulations, 80 participants were recruited for this study (40 per group).

This study updated the screening method for honesty tendencies, with participants being screened for individual personality traits and short-term decision-making. First, a cluster sampling method was used to select three high schools in Lianyungang City and Xuzhou City, both in Jiangsu Province, as well as Tianjin. The honesty–humility subscale of the Chinese version of the HEXACO-100 Personality Scale (Ashton et al., 2004) was used to assess 500 adolescents, resulting in 481 valid questionnaires from 196 boys and 285 girls. According to the total average score of the questionnaire, 15% before and after ranking were defined as the low- honesty-humility group (lower than 2.31) and the high-honesty-humility group (higher than 3.94), respectively. A total of 144 individuals with high and low honesty tendencies were screened for participation. The selected participants were then subjected to a dice-rolling task, wherein their (dis)honest behavioral choices were used as a measure of short-term behavioral decisions. Finally, participants in the high honesty–humility group who displayed honesty in the dice-rolling task were selected as the high honesty tendency group, while those who were in the low honesty–humility group and displayed dishonesty in the dice-rolling task were selected as the low honesty tendency group for the formal experiment. Finally, 80 valid participants (40 with high and 40 with low honesty tendencies) were selected, including 32 boys and 48 girls, with an average age of 16.61 ± 1.54 years.

The inclusion criteria and the consent and ethical approval processes were the same as in Pre-Experiments 1 and 2.

#### 3.2.2. Experimental Design

The experiment adopted a 2 (honesty tendency: high vs. low) × 2 (decision-making mode: intuitive vs. deliberate) × 2 (decision-making situations: victim vs. victimless) mixed experimental design, considering honesty tendency as a between-subjects variable, and decision-making modes and situations as within-subjects variables. Cognitive load and time constraint were used to manipulate the decision-making mode, with deception rate in the “spot-the-difference” task as the dependent variable.

#### 3.2.3. Experimental Materials and Tasks

Honesty–Humility: The honesty–humility subscale of the Chinese version of the HEXACO-100 personality scale was used to measure participants’ honesty–humility level. The scale comprises four dimensions—sincerity, fairness, modesty, and greed-avoidance—with a total of 16 items (e.g., “If I am sure I will never be caught, I will be tempted to use counterfeit money”) rated on a 5-point Likert scale (1 = totally disagree, 5 = totally agree), with higher scores indicating higher honesty–humility levels. The scale has been proven to have good reliability and validity in both adolescents and adults (Garbe et al., 2020; Guo et al., 2023). In the experiment, the Cronbach’s alpha reliability of the honesty–humility subscale was 0.89, indicating high internal consistency.

Dice-rolling task: This task was used to examine the characteristics of participants’ short-term honest decision-making. This was combined with the honesty–humility scale to screen participants with high and low honesty tendencies who participated in the formal experiment. The core design of this task includes an autonomous reporting mechanism and covert program control (Dubois et al., 2015). The task involved asking participants to roll five consecutive dice by pressing the space bar on a computer and to report their cumulative total score at the end. When the total score declared by the participants reached or exceeded 14 points, they received a 20 yuan gift card as a reward. Participants performed the dice-rolling actions independently throughout the process but had to record the results of each roll. During the experiment, the experimenter temporarily left the laboratory, creating an unsupervised environment in which potential dishonest behaviors could be observed. Notably, the program restricted the total number of dice points to no more than 12 points through the algorithm. This hidden setting that allowed participants to report their results as exceeding 12 points was directly identified as dishonest behavior, whereas a report of ≤12 points was considered an objective criterion for honest behavior.

Spot-the-difference task: This formal experimental task was used to measure honest behavior. Participants were presented with image pairs on a computer and informed that there were three differences between the image pairs. However, unlike the pre-experiment, the images in the formal experiment could contain one, two, or three differences. Participants were rewarded if they simply reported that they found the three differences in the images, without having to specify the three different locations. This design provided participants with the opportunity to cheat during the test. If participants honestly reported not finding three differences in image pairs with fewer than three differences, it was considered honest behavior. The images with three differences were classified as normal trials—with a reward amount of 0.25 yuan. Images with one or two differences were randomly classified as difficult trials—with a reward of 1 yuan—or very difficult trials—with a reward of 2 yuan. Different difficulty levels were introduced to minimize participants’ suspicions about the true purpose of the task. In reality, no significant difference existed in difficulty between the image pairs. The hypothesis was that if the images were labeled as difficult or very difficult, it would be more credible to the participants that the image pair actually contained three differences, but they were just too difficult to identify (Speer et al., 2020). In addition, difficulty ratings were introduced to eliminate possible demand effects. The study sought to encourage participants to cheat for monetary rewards rather than to prevent them from seeming incompetent.

To prevent limitations in perception and attention arising from the inherent difficulty of the image materials, this study rigorously screened the experimental materials through a pre-experiment. The pre-experiment results showed that 95% of the participants found all three differences in less than 4000 ms. This high success rate indicated that the ambiguity and difficulty of the differences in images was low. Consequently, the probability of false positives was minimized, ensuring that the behavioral responses observed in the experiment more accurately reflected participants’ moral intentions.

Two-response paradigm: A two-response paradigm was used to manipulate dual-processing. This paradigm separates individual decision-making modes into intuitive and deliberate decision-making, aligning with decision-making situations in the living environment. The paradigm is considered to offer the most direct evidence for evaluating the dual-processing theory (Thompson & Johnson, 2014; Y. H. Wang & Luo, 2024; Yang & Chen, 2020). This paradigm involves the cognitive load task (dot memorization task) and formal experimental task (spot-the-difference task) being performed simultaneously. First, the participants were asked to perform a dot memorization task, then a visual search task, before answering the presented question. The answer must be the first thought that comes to mind. Participants were then asked to perform dot memorization recognition. Performing both tasks simultaneously consumes a significant amount of cognitive resources (high cognitive load), thereby maximizing the likelihood that participants’ first response to the question is an intuitive decision. When the same question was presented again, participants were told that they could contemplate the question without time limitations and then provide an answer (low cognitive load). Therefore, the second answer to the same question can be regarded as more inclined to use deliberate, rational decision-making.

A single decision-making mode manipulation method may be affected by a specific experimental setting or subject group. For example, when manipulating the decision-making mode only through time constraints, subjects may still think carefully before setting time pressure (Shi et al., 2020). Although decision time can partially validate manipulation effectiveness in some studies (Capraro et al., 2019; Hausladen & Nikolaychuk, 2024), uncertainty remains. Employing multiple manipulations synergistically can overcome the limitations of single manipulations and enhance the reliability of research findings (Mischkowski et al., 2018; Shi et al., 2024). To clearly distinguish between intuitive and deliberate decision-making modes, this study adopted a more rigorous approach. It combined cognitive load with time constraints, requiring participants to respond under a certain time constraint while performing the dot memorization load task. By restricting thinking time and depleting cognitive resources, the study ensured the validity of decision-making mode manipulation.

#### 3.2.4. Experimental Procedure

After arriving at the laboratory, participants with different honesty tendencies were placed in quiet, independent rooms. After understanding the process of the experiment, participants signed an informed consent form and began the experiment. The program was written using E-Prime 3.0.

The experiment involved complex visual search tasks (dot memorization and spot-the-difference tasks). First, participants were told that there were two rounds of the task to complete. The first round involved completing the task independently, and the second round involved completing the task with the others. There were 48 image pairs in each round. Victim situations were set up by manipulating the allocation of money (Amir et al., 2016; Pitesa et al., 2013; Soraperra et al., 2018). In victim situations (completing the task with others), the participants were told: “You and the other party will receive your respective rewards from a fixed total. The more you receive, the less the other party receives. Thus, your dishonesty will harm others. The initial amount for both parties is 0 yuan. Pressing “yes” indicates that you have found three differences in the image and are therefore liable to receive the rewards for this round, with the other party receiving 0 yuan. Pressing “no” means that you have not found three differences in the image, meaning that you will receive 0 yuan, with the other party winning this round.” In victimless situations (completing the task independently), participants had to press “yes” to receive and “no” to lose the rewards in each round. The victim/victimless situations were presented equally among participants.

To ensure the authenticity of the interaction, two participants (one of whom was an experimental assistant) arrived at the laboratory simultaneously. After greeting each other, they had to complete the task in separate rooms. Then, the participants were informed, “according to the task rules, there are two roles in the experiment: the decision maker and the receiver. It is necessary to decide who is the decision maker by drawing lots.” In fact, the lots were programmed so that participants could only select the decision-maker’s role and decide on money allocation.

**Figure 2:**
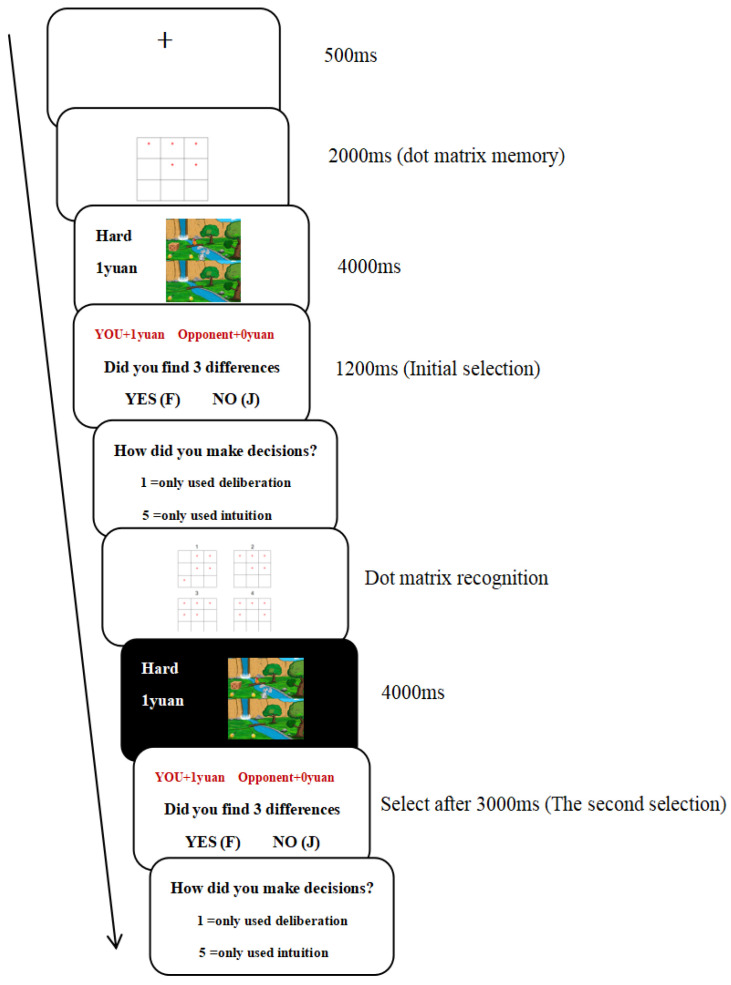
Flowchart of the two-response paradigm in victim situations.

The specific procedure for victim situations was as follows. First, a fixation point appeared on the screen for 500 ms, followed by a nine-square grid. Participants were required to remember the positions of the five asterisks (*) in the grid (2000 ms, dot matrix memory). Subsequently, a pair of similar images was presented on the screen, each pair containing three differences. Simultaneously, the screen displayed the difficulty level of the images and the reward amount for the trial on a white background (4000 ms). Immediately afterward, the screen displayed the prompt “Did you find 3 differences?” The participants were required to respond quickly, based on intuition, within 1200 ms. Selecting “Yes” by pressing the “F” key would grant them the reward, with the screen displaying “You: X yuan, Opponent: 0 yuan.” Selecting “No” by pressing the “J” key would forfeit the reward, with the screen displaying “You: 0 yuan, Opponent: X yuan.” Then, participants were asked to evaluate how they made decisions in their current decision-making mode (1 = only used deliberation, 5 = only used intuition). Next, four nine-square grids were displayed for the dot matrix recognition task. The screen indicated whether the recognition results were correct. The screen then displayed the same image pairs that appeared earlier with a black background (4000 ms). Subsequently, the screen displayed the prompt, “Did you find 3 differences? Please think carefully for 3000 ms before responding. Selecting ‘Yes’ and pressing the ‘F’ key would earn you a reward, whereas selecting ‘No’ and pressing the ‘J’ key would result in forfeiting the reward. If ‘Yes’ is selected, the screen will display ‘You: X yuan, Opponent: 0 yuan’. If ‘No’ is selected, the screen will display “You: 0 yuan, Opponent: X yuan.” We then evaluated how decisions were made following the current decision-making process (1 = only used deliberation, 5 = only used intuition). A 500 ms fixation point was then presented, and the next trial was initiated. A flowchart of this process is illustrated in Figure 2.

At the end of the experiment, participants’ payoffs were displayed on the screen. After the first round of tasks, participants rested for three minutes, following which the second round of tasks began, involving victimless situations. The experimental process was the same as that of the first round, except that the reward amount rule in the victimless situation involved only oneself. The formal experiment consisted of 96 trials, divided into two rounds of 48 trials each. Four practice trials were conducted. After completing the experiment, participants were informed of its true purpose. To ensure fairness, they all received the maximum reward regardless of their actual level of dishonesty.

#### 3.2.5. Data Analyses

Considering that the three-factor mixed experimental design includes both between-subjects variables (honesty tendency) and within-subjects variables (decision-making modes, victim situations), the 2 × 2 × 2 repeated measures analysis of variance (ANOVA) was an appropriate method for testing its main effects and interactions. This method can effectively control for individual differences and effectively examine how decision-making modes and victim situations affect the cheating rate of adolescents with different honesty tendencies. Data analysis and processing were conducted using SPSS (version 27.0; IBM Corporation, New York, NY, USA) software.

### 3.3. Results

#### 3.3.1. Manipulation Checks for Decision-Making Modes

In the two-response paradigm, the accuracy of the memory matrix was more than 85%, indicating that participants had invested considerable cognitive resources in the dot matrix memory task. Simultaneously, the paired sample *t*-test results revealed a significant difference in the decision-making mode used by the participants under high/low cognitive load conditions, *t* (79) = 15.44, *p* < 0.001, Cohen’s *d* = 0.85. Participants reported using intuitive decision-making more frequently under high-cognitive load conditions (*M* = 3.67, *SD* = 0.49) than under low-cognitive load conditions (*M* = 2.23, *SD* = 0.65), implying that manipulating decision-making modes was effective.

#### 3.3.2. Normal Distribution Test

Before repeated measures ANOVA, the Shapiro–Wilk test was used to test the normality of the deception rate of high and low honesty tendency adolescents across the following conditions: intuitive mode in victim situations, deliberate mode in victim situations, intuitive mode in victimless situations, and deliberate mode in victimless situations. The results showed that adolescents with low honesty tendency had a normal distribution of deception rates in the deliberate mode in victim situations (*p* = 0.25), the intuitive mode in victimless situations (*p* = 0.376), and the deliberate mode in victimless situations (*p* = 0.248). However, the deception rate in the intuitive mode in victim situations (*p* = 0.001) did not fit to a normal distribution. The deception rates of adolescents with high honesty tendency in the intuitive mode in victim situations (*p* = 0.021), the deliberate mode in victim situations (*p* = 0.033), and the deliberate mode in victimless situations (*p* = 0.035) did not fit to a normal distribution, while the deception rate in the intuitive mode in victimless situations (*p* = 0.332) was fit to a normal distribution.

As the repeated measures ANOVA for this study was based on a large sample size (*N* = 80), and the sample sizes were balanced across the groups, although the original data distribution of some variables deviates from the normal distribution, according to the central limit theorem, the distribution of sample means tends to approach the normal distribution in the case of large samples (Field, 2018). At the same time, previous studies generally believe that ANOVA is robust to violations of normality assumptions (Field, 2018; Glass et al., 1972). Therefore, repeated measures ANOVA was considered appropriate for this study.

#### 3.3.3. Deception Rate

Mauchly’s Test of Sphericity was performed on decision-making mode, victim situations, and decision-making mode × victim situation interactions. The results showed that the *p*-values of the sphericity test of all variables were less than 0.05, indicating that the data did not satisfy the sphericity test. Because epsilon (ε) > 0.75, *p*-values were corrected by the Huynh–Feldt method.

A 2 (honesty tendency: high vs. low) × 2 (decision-making mode: intuitive vs. deliberate) × 2 (decision-making situations: victim vs. victimless) repeated measures ANOVA was performed on the cheating rate. The results are presented in Table 1.

The results revealed that the main effect of honesty tendencies was significant: *F*(1, 78) = 251.89, η_p_^2^ = 0.76, *p* < 0.001. The cheating rate of adolescents with low honesty tendency was significantly higher than that of those with high honesty tendency. The main effect of decision-making modes was significant: *F*(1, 78) = 4.36, η_p_^2^ = 0.05, *p* < 0.05. The deception rate of the intuitive decision-making mode was significantly lower than that of the deliberate decision-making mode. The main effect of victim situations was significant: *F*(1, 78) = 153.50, η_p_^2^ = 0.66, *p* < 0.001. The deception rate in victim situations was significantly lower than that in victimless situations.

A significant interaction was observed between honesty tendency and decision-making modes: *F*(1, 78) = 10.32, η_p_^2^ = 0.12, *p* < 0.01. A simple effects analysis revealed that the deception rate of adolescents with high honesty tendency in the intuitive decision-making mode was significantly lower than that in the deliberate decision-making mode: *F*(1, 78) = 14.04, η_p_^2^ = 0.15, *p* < 0.001. No significant difference was observed between the deception rates of adolescents with low honesty tendency in the intuitive and deliberate decision-making modes: *F*(1, 78) = 0.63, *p* = 0.429.

The interaction between honesty tendency and victim situation was significant: *F*(1, 78) = 13.48, η_p_^2^ = 0.15, *p* < 0.01. A simple effects analysis revealed that the cheating rate of adolescents with high honesty tendency in the victim situation was significantly lower than that in the victimless situation: *F*(1, 78) = 128.98, η_p_^2^ = 0.62, *p* < 0.001. The deception rate of adolescents with low honesty tendency was significantly lower in the victim than in the victimless situation: *F*(1, 78) = 38.00, η_p_^2^ = 0.33, *p* < 0.001. This indicates that adolescents with high and low honesty tendencies tend to behave more honestly in victim situations.

The interaction between the decision-making mode and victim/victimless situation was significant: *F*(1, 78) = 35.96, η_p_^2^ = 0.32, *p* < 0.001. A simple effects analysis revealed that in victim situations, the deception rate under the intuitive decision-making mode was significantly lower than that under the deliberate decision-making mode: *F*(1, 78) = 33.03, η_p_^2^ = 0.30, *p* < 0.001. In victimless situations, the deception rate in the intuitive decision-making mode was significantly higher than that in the deliberate decision-making mode: *F*(1, 78) = 8.55, η_p_^2^ = 0.10, *p* < 0.01.

**Figure 3:**
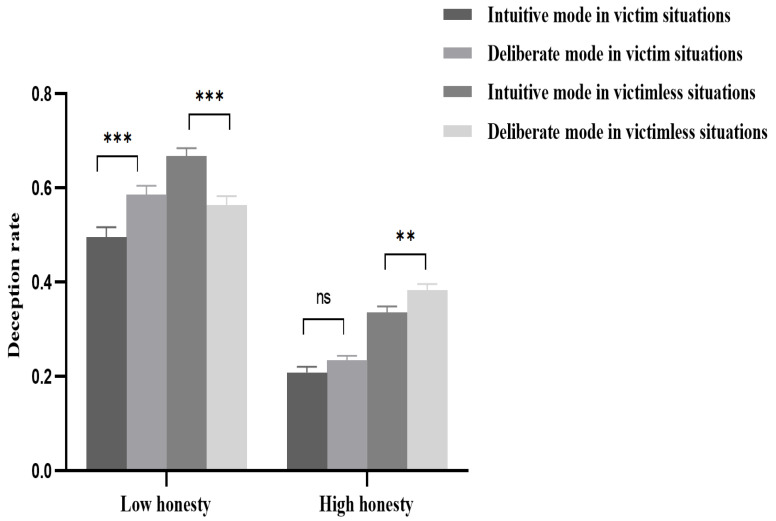
The effect of decision-making modes in victim/victimless situations on the honest behaviors of adolescents with high and low honesty tendencies. ***: *p* < 0.001; **: *p* < 0.01; ns: not significant, *p* > 0.05.

The interaction effects of honesty tendencies, decision-making modes, and victim situations were significant: *F*(1, 78) = 54.35, η_p_^2^ = 0.41, *p* < 0.001. Further analysis revealed that for adolescents with low honesty tendency, the main effect of decision-making modes was significant in victim situations: *F*(1, 78) = 39.20, η_p_^2^ = 0.33, *p* < 0.001. Specifically, the deception rate under the intuitive decision-making mode was significantly lower than that under the deliberate decision-making mode. In victimless situations, the main effect of decision-making modes was also significant: *F*(1, 78) = 55.41, η_p_^2^ = 0.42, *p* < 0.001. Specifically, the deception rate under the intuitive decision-making mode was significantly higher than that under the deliberate decision-making mode. For adolescents with high honesty tendency, the main effect of decision-making modes was not significant in victim situations—*F*(1, 78) = 3.48, *p* = 0.066—indicating that the difference in deception rates between intuitive and deliberate decision-making was not significant. In victimless situations, the main effect of the decision-making mode was significant—*F*(1, 78) = 10.95, η_p_^2^ = 0.12, *p* < 0.01—with the deception rate of intuitive decision-making being significantly lower than that of deliberate decision-making. The results are shown in Figure 3.

## 4. Discussion

This study explored the dynamic changes in honest behaviors between adolescents with high and low honesty tendencies under intuitive and deliberate decision-making modes from the perspective of individual–situational interaction. The results revealed that the influence of decision-making modes on honest behaviors was affected by not only individual traits of honesty tendencies but also victim situational factors. Specifically, in victimless situations, the influence of decision-making modes on honest behaviors differed between adolescents with high and low honesty tendencies. Promoting intuitive decision-making was more conducive to adolescents with high honesty tendency engaging in more honest behaviors, whereas promoting deliberate decision-making was more conducive to adolescents with low honesty tendency engaging in more honest behaviors. In victim situations, the influence of decision-making modes on honest behaviors tended to be consistent between adolescents with high and low honesty tendencies, who exhibited more honest behaviors in the intuitive decision-making mode. The results indicated that victim situational factors played a very important role in how decision-making modes affected adolescents’ honest behaviors.

This study extends previous studies that examined individual traits and situational factors in isolation. It emphasizes the dynamic, interactive nature of honesty behavior—it is neither completely determined by individual traits nor solely governed by situational factors, but a dynamic process shaped by both. This interactive framework not only provides a powerful explanation for reconciling conflicting findings, but also enriches and expands the application of the social heuristic hypothesis in honesty research.

### 4.1. Honesty Tendencies and Decision-Making Modes Influence Adolescents’ Honest Behaviors in Victimless Situations

This study found that in victimless situations, the influence of decision-making modes on adolescents’ honest behaviors was moderated by honesty tendencies. For adolescents with low honesty tendencies, dishonesty was their default moral response, and promoting intuitive decision-making would lead to more dishonest behavior. Therefore, honest behaviors resulted from deliberate decision-making, confirming the “Will” hypothesis. For adolescents with high honesty tendency, honest behaviors were more likely to result from intuitive decision-making, confirming the “Grace” hypothesis. Thus, the decision-making mode that promotes honest behaviors depends on the individual’s own honesty tendency (Speer et al., 2023; Warner et al., 2022). The results revealed that this trait was an important source of the heterogeneity in dishonest behaviors (Speer et al., 2020, 2021). This reconciles the debate in previous studies in behavioral research and further confirms that both the “Will” and “Grace” hypotheses have partial explanatory power (Speer et al., 2020, 2021). However, individual honesty tendencies should be combined to make conditional corrections for the two hypotheses.

In line with the SHH, honest behaviors among adolescents with high honesty tendency can be understood as default, intuitive responses shaped by successful social strategies. This intuitive response is repeatedly reinforced in familiar social situations so that individuals will automatically use it to make decisions when faced with new and unfamiliar situations (such as laboratory environments) (Rand et al., 2014). Adolescents are influenced by family upbringing, school education, peer groups, and their social environment as they grow up. Most adolescents have a strong sense of identification with good moral qualities such as honesty, fairness, and integrity (Vadera & Pathki, 2021). Adolescents with high honesty tendency, especially, place greater emphasis on the long-term benefits of honesty, such as a good reputation and mutually trusting interpersonal relationships (Timpau, 2015; Malti et al., 2021). When the surrounding environment values honesty and peers demonstrate trustworthiness, adolescents with high honesty tendency show stronger motivation to maintain honest behavior. In this context, honesty gradually becomes their moral default. For them, honest behavior is the result of the intuitive decision-making mode.

Beyond external reinforcement, an emphasis on a positive self-concept constitutes another crucial factor promoting intuitive honest behaviors among adolescents with high honesty tendency (Speer et al., 2020). Studies have found that adolescents with high honesty tendency have a higher moral bottom line and usually avoid engaging in fraud, theft, or deception. Compared with the temptation of monetary interests, they attach greater importance to preserving their internal moral self-concept (Zettler et al., 2020). In the course of socialization, this orientation facilitates the gradual internalization of positive social norms and moral values that constitute the standards for self-evaluation. When the behavior of adolescents with high honesty tendency conforms to social rules (e.g., honesty, altruism, cooperation), they are rewarded by their internal reward system, experiencing improved self-esteem, gaining trust and respect from others, and establishing stable interpersonal relationships. On the contrary, cheating and rule-breaking violate their moral standards, triggering punishment from their internal system (e.g., guilt, shame) and forcing a negative update of their self-concept (Speer et al., 2020). Therefore, higher moral standards and an emphasis on a positive self-concept are important reasons for adolescents with high honesty tendency to engage in more honest behaviors under the intuitive decision-making mode.

In contrast, in daily life, some teenagers regard dishonesty as their default moral behavior. Adolescence is a critical period for moral development. Owing to the immaturity of self-control, adolescents are particularly vulnerable to external environmental influences. Especially under the impact of a materialistic value orientation, some adolescents pay more attention to material wealth and prioritize the pursuit of personal interests (Timpau, 2015). Such values may undermine their honest decision-making behavior. Evidence indicates that individuals low in honesty–humility are typically driven by material gains to ingratiate others or violate rules (De Vries et al., 2017). When these behaviors result in personal benefits, adolescents show a greater propensity for unethical behaviors (MacDonell & Willoughby, 2020; Volk et al., 2018). Experimental research further demonstrated that low-honesty–humility individuals consistently displayed higher levels of dishonesty in tasks such as coin-tossing and dice-rolling (Hilbig & Zettler, 2015). Consequently, adolescents with low honesty tendency not only practice deceit more frequently but also refine strategies of moral disengagement and develop self-serving rationalizations for lying (Sijtsema et al., 2019; Guo et al., 2021). Heightened moral disengagement, in turn, facilitates deception, rendering dishonest responses increasingly automatic (Guo et al., 2021). Deception has become a relatively automatic and dominant response for adolescents with low honesty tendency. Under the intuitive decision-making mode, adolescents with low honesty tendency were more likely to engage in default deceptive behaviors. However, under the deliberate decision-making model with sufficient cognitive resources, they can effectively suppress impulses and temptations and generate moral consciousness (Speer et al., 2020). Only in this way can they re-evaluate their behaviors and adjust their behavioral decisions accordingly, as they no longer focus solely on short-term benefits but consider the long-term impact of their actions. Consequently, in the deliberate decision-making mode, they are more likely to choose honest behaviors that align with moral standards and social expectations.

In conclusion, whether honest behavior originates from intuitive or deliberate decision-making modes depends on an individual’s inherent moral default. For adolescents whose moral defaults lean toward honesty, the intuitive decision-making mode tends to foster honest behaviors. Conversely, for those whose moral defaults lean toward dishonesty, the deliberate decision-making mode is often more instrumental in promoting honest behaviors.

These findings suggest that integrity education should be differentiated based on honesty predisposition to shift moral guidance from “standardized production” to “personalized cultivation.” For adolescents with low honesty tendency, educational interventions could follow a progressive “external control to internalization” transformation pathway. Through intervention strategies such as behavior shaping and cognitive restructuring, the focus centers on cultivating a sense of moral responsibility and self-restraint capabilities, with the aim of enhancing their identification with and intrinsic motivation for honest behavior, facilitating the transition from behavioral conditioning to value reconstruction. For adolescents with high honesty tendency, the educational emphasis should focus on consolidating their existing honest behaviors while enhancing their moral resilience and contextual resistance (L. Y. Wang et al., 2024). This encourages them to maintain consistent honest conduct across diverse situations, thereby preventing their established moral traits from deteriorating in complex environments. Educators can also encourage adolescents with high honesty tendency to take on the role of peer mentors in the classroom, making them “disseminators” of honest behavior among their peers. By externalizing responsibility (e.g., serving as a monitor team leader), their self-identity is consolidated, creating a virtuous cycle of moral exemplification and expanding the influence of their honest behaviors.

### 4.2. Honesty Tendencies and Decision-Making Modes Influence Adolescents’ Honest Behaviors in Victim Situations

In victim situations, the influence of decision-making modes on adolescents’ honest behaviors was shaped by both individual differences and situational dependency. The results revealed that adolescents with high honesty tendency exhibited relatively stable honest behaviors in victim situations. Adolescents with low honesty tendency also exhibited more honest behaviors in victim situations when intuitive decision-making was promoted. This finding suggests that adolescents with low honesty tendency are not consistently dishonest. When the situational cues indicating harm to others are activated, their intuitive responses tend toward honesty. It further indicated that the intuitive moral responses of adolescents with low honesty tendency are not fixed and unchangeable, but change dynamically according to the situation. Situational cues (i.e., the presence of a victim) can temporarily override an individual’s inherent default moral tendency (Köbis et al., 2019; Shi et al., 2020).

It should be noted that some eta-square values in the study were unusually large. This may be attributed to two factors: first, the study employed a rigorous grouping procedure combining personality scales with behavioral screening, which significantly amplified intergroup differences (high/low honesty tendencies). Second, the experimental task (the spot-the-difference task) provided a clear opportunity to cheat, and the manipulation of the victim situations had a powerful moral priming effect. These design features collectively enhanced the explanatory power of the independent variable on honest behavior, consistent with expectations from studies examining robust individual differences in highly controlled laboratory settings.

These findings are similar to those of previous studies and support the view that considering the negative impact of one’s own unethical behavior on others reduces the likelihood of acting in selfish and dishonest ways (Köbis et al., 2019; Meub et al., 2016; Soraperra et al., 2018). Prior research has revealed that the decision to be honest primarily depends on two aspects: the magnitude of the (external or internal) reward gained from lying and the degree of harm caused to others by the lie (respect for others) (Gneezy, 2005). Individuals care not only about their own welfare but also about whether others experience gains or losses. Thus, regardless of their high or low honesty tendency, when individuals’ decisions affect others, they continuously weigh and adjust their own benefits against the costs imposed on others (Barneron et al., 2021) and tend to exhibit more honest behaviors.

This phenomenon can be explained by the theory of prosocial instincts in evolutionary psychology and the drive of moral emotion. According to Haidt’s (2001) social intuition theory, humans have developed a prioritized sensitivity to harm avoidance during evolution. This moral intuition—serving as an adaptive mechanism—can rapidly identify scenarios that potentially cause suffering for others and trigger protective behavioral responses. In this process, moral emotion, represented by guilt, plays an important role. Studies have found that anticipatory guilt can act as a prospective emotional motivation to act honestly (Tangney et al., 2007; Cohen et al., 2012). Reactive guilt, on the other hand, drives individuals to repair moral self-concept and relieve emotional discomfort through compensatory prosocial behaviors (such as honesty) after inappropriate behavior (Motro et al., 2018; Tang et al., 2019; Sznycer, 2019). It was evident that guilt emotions can promote the internalization of morality, making self-control and moral standards stricter, and thereby influencing people’s behavioral decisions. The latest research by Gangemi et al. (2025) also found that moral emotions, such as guilt, can influence an individual’s preference for risk choices based on their moral goals (reparation or expiation), highlighting the significant role of moral emotions in driving behavioral decisions. There were also studies that explained it at the level of neural mechanisms. Previous research has revealed that when individuals are confronted with cues of victim suffering, the anterior insula and amygdala exhibit significant activation within 200–400 ms (Decety & Cacioppo, 2012). The immediacy of this neural response far precedes the speed of cognitive evaluation by the prefrontal cortex, which serves as the neural basis for intuitive moral decision-making. Previous studies have found that adolescents’ limbic systems develop earlier than do their prefrontal control networks (Steinberg, 2008), suggesting that their affect-driven systems have stronger reactivity to victim situations. This makes the intuitive decision-making mode based on moral emotions more important in behavioral decision-making (Meldrum et al., 2018). Dickert et al. (2011) found that compared with vague and abstract victims, concrete victims elicited stronger associations, conjectures, and mental representations of others’ situations in decision-makers. Furthermore, compared to unidentifiable victims, losses and harm to identifiable victims evoke more intense emotional experiences in individuals, such as empathy and guilt. This, in turn, motivates decision-makers to engage in more prosocial behaviors toward others (Hou et al., 2023; Zhao et al., 2024). Therefore, the automatic arousal of moral affect may explain why adolescents with both high and low honesty tendencies are more honest under the intuitive decision-making mode in victim situations.

An alternative explanation for this result is that victim situations typically have high situational clarity, and one’s choices directly harm the interests of others. This explicit behavioral consequence makes it harder for individuals with low honesty tendency to find excuses to rationalize their dishonest behaviors in the intuitive decision-making mode. Situational clarity reduces opportunities for moral disengagement, thereby prompting adolescents with low honesty tendency to choose more honest behaviors under the intuitive decision-making mode (Barneron et al., 2021; Lee & Feeley, 2016). These findings are also consistent with the “do no harm” principle, which posits that individuals are unwilling to harm others for their own benefit (Y. Zhang et al., 2022), because decisions that cause loss to others are perceived as a form of harm. This heightens individuals’ concern for others through their aversion to harm (Thunström, 2019). Research has revealed that framing unethical behaviors as more aggressive can increase the internal cost of engaging in such acts and deter dishonest behaviors (Mazar et al., 2008). Although adolescents with low honesty tendency have higher greed motivation, increasing personal gain by harming others’ interests violates the “do no harm” principle and entails significant psychological costs. Explicit victims are better able to evoke strong emotional reactions, such as guilt, self-blame, and a sense of responsibility (Meub et al., 2016). Consequently, the higher moral cost is also a crucial reason why the moral intuition of adolescents with low honesty tendency is more inclined toward honesty in situations involving harm to others (Shi et al., 2020).

These findings indicate that moral education cannot merely focus on the value indoctrination of “honesty is a virtue.” It needs to address the complexity of moral decision-making in real-world situations. This study highlighted that honest behavior is strongly situationally dependent. Indeed, the presence of harm to others emerged as a particularly critical factor. This suggests that, in daily life, individuals should pay greater attention to how activating awareness of the consequences of dishonest behaviors in a given context can positively influence honesty (Köbis et al., 2019). Situational cues (such as enhancing the visibility of behavioral consequences) can be embedded in institutional design to improve overall societal integrity levels. Changing an individual’s honest personality trait is difficult in the short term. However, we can shape their preference for honesty through specific methods (Huber et al., 2023), including altering decision-making modes, designing norms for moral behavior, and optimizing situations. This approach offers an effective path toward greater honesty. This has implications for fostering honesty and building a harmonious society in the long run.

### 4.3. Limitations and Future Directions

First, this study focused solely on mid–late Chinese adolescents. Future research should expand the scope of participant groups to include diverse cultural backgrounds, school settings, genders, and socioeconomic strata (Cohn et al., 2019; Gächter & Schulz, 2016; Kanngiesser et al., 2024; Keller & Kiss, 2025). This would help further improve the ecological validity of the research, expand the applicability of the conclusions, and supplement and enrich the present results.

Second, concerning individual differences, this study primarily examined the influence of the honesty–humility personality trait. Future studies should consider other personality traits reflecting prosocial tendencies, such as virtuous personality traits and social value orientation (H. Y. Zhang et al., 2021; Grosch & Rau, 2017). Furthermore, future research should refine the moderating factors related to individuals’ implicit preferences and cognitive processes to identify those that better predict individual behaviors.

Third, regarding situational factors, this study focused primarily on the victim/victimless situations, without further subdividing victim situations. Does the mere presence of a victim necessarily promote honest behavior? Is the victim effect consistently stable? Future research could explore the impact of different victim types (in-group vs. out-group) and victim loss–gain contexts on honest behaviors in a similar manner (Bilancini et al., 2020; Nagel et al., 2021). This would enable a more comprehensive understanding of the role of victim situations in the relationship between decision-making modes and honest behaviors.

Fourth, this study only focused on personality traits and situational factors, and did not further investigate the role of moral-emotional factors. Future research can incorporate the measurement and manipulation of moral emotions to investigate the changes in honest behavior among adolescents under different decision-making modes under different conditions of moral emotion initiation, such as guilt and shame, deepening the three-dimensional theoretical framework of “personal traits–situations–moral emotional mechanism” to provide a more solid scientific basis for understanding and promoting the moral development of adolescents.

Fifth, this study primarily examined the effect of decision-making patterns on honest behaviors from a behavioral perspective, failing to investigate its underlying neural mechanisms. Capturing the internal psychological process in complex social decision-making is difficult in behavioral research. Therefore, future research should combine various neuroscience techniques (Li et al., 2022) to reveal the intrinsic neural mechanisms of decision-making patterns affecting honest behaviors to further clarify the characteristics of cognitive neural activation of individuals with different honesty tendencies in the process of honest decision-making. This would provide clearer and more compelling evidence to reconcile research debates from a neural mechanism perspective.

## 5. Conclusions

This study investigated the influence of decision-making modes on adolescents’ honest behaviors from an individual–situation interaction perspective. The results revealed that the effect of decision-making modes on adolescents’ honest behaviors was moderated by individual traits (honesty tendency) and situational factors (victim presence). Specifically, in victimless situations, promoting intuitive decision-making facilitated more honest behaviors among adolescents with high honesty tendencies, whereas promoting deliberate decision-making encouraged more honest behaviors among those with low honesty tendencies. In victim situations, when dishonest behaviors caused harm to others, adolescents with both high and low honesty tendencies engaged in more honest behaviors under the intuitive decision-making mode.

## Figures and Tables

**Table 1:** Deception rates of adolescents with high and low honesty tendencies in intuitive and deliberate decision-making modes in victim/victimless situations.

Honesty Tendency	Decision-Making Mode	Victim Situations		Victimless Situations	
		*M*	*SD*	*M*	*SD*
high (*n* = 40)	intuitive	0.21	0.08	0.34	0.08
	deliberate	0.23	0.06	0.38	0.08
low (*n* = 40)	intuitive	0.49	0.13	0.67	0.11
	deliberate	0.58	0.12	0.56	0.12

## Data Availability

The data will be available upon request.

## Abbreviations

The following abbreviations are used in this manuscript:

ANOVA	Analysis of variance
HEXACO	Honesty–humility, Emotionality, Extraversion, Agreeableness, Conscientiousness, and Openness to experience
M	Mean
SHH	Social heuristic hypothesis
SD	Standard deviation
